# Supplementary material for: HIVprotI: an integrated web based platform for prediction and design of HIV proteins inhibitors
Source: J Cheminform. 2018 Mar 9;10:12. doi: 10.1186/s13321-018-0266-y (PMC5845081; doi:10.1186/s13321-018-0266-y)
Supplement: Supplementary file 2 — Additional file 2. Source code of HIVProtI web server. [file 13321_2018_266_MOESM2_ESM.zip › HIVprotI_Source-code/hivprotI/weka-3-6-6/doc/weka/classifiers/meta/MultiScheme.html]

MultiScheme


---


|  |  |  |
| --- | --- | --- |
| **Overview** \| **Package** \| **Class** \| **Tree** \| **Deprecated** \| **Index** \| **Help** \| **Weka's home** | |  |
| **PREV CLASS**   **NEXT CLASS** | **FRAMES**    **NO FRAMES**     **All Classes** |  |
| SUMMARY: NESTED \| FIELD \| CONSTR \| METHOD | DETAIL: FIELD \| CONSTR \| METHOD |  |


---


## weka.classifiers.meta Class MultiScheme

```
java.lang.Object
  weka.classifiers.Classifier
      weka.classifiers.MultipleClassifiersCombiner
          weka.classifiers.RandomizableMultipleClassifiersCombiner
              weka.classifiers.meta.MultiScheme
```

**All Implemented Interfaces:**: java.io.Serializable, java.lang.Cloneable, CapabilitiesHandler, OptionHandler, Randomizable, RevisionHandler

---

``` public class MultiScheme extends RandomizableMultipleClassifiersCombiner ```

Class for selecting a classifier from among several using cross validation on the training data or the performance on the training data. Performance is measured based on percent correct (classification) or mean-squared error (regression).


Valid options are:

```
 -X <number of folds>
  Use cross validation for model selection using the
  given number of folds. (default 0, is to
  use training error)
```

```
 -S <num>
  Random number seed.
  (default 1)
```

```
 -B <classifier specification>
  Full class name of classifier to include, followed
  by scheme options. May be specified multiple times.
  (default: "weka.classifiers.rules.ZeroR")
```

```
 -D
  If set, classifier is run in debug mode and
  may output additional info to the console
```

**Version:**
:   $Revision: 1.25 $

**Author:**
:   Len Trigg (trigg@cs.waikato.ac.nz)

**See Also:**: Serialized Form

---

**Constructor Summary**

`MultiScheme()`


| **Method Summary** | |
| --- | --- |
| `void` | `buildClassifier(Instances data)`             Buildclassifier selects a classifier from the set of classifiers by minimising error on the training data. |
| `java.lang.String` | `classifiersTipText()`             Returns the tip text for this property |
| `java.lang.String` | `debugTipText()`             Returns the tip text for this property |
| `double[]` | `distributionForInstance(Instance instance)`             Returns class probabilities. |
| `int` | `getBestClassifierIndex()`             Get the index of the classifier that was determined as best during cross-validation. |
| `Classifier` | `getClassifier(int index)`             Gets a single classifier from the set of available classifiers. |
| `Classifier[]` | `getClassifiers()`             Gets the list of possible classifers to choose from. |
| `boolean` | `getDebug()`             Get whether debugging is turned on |
| `int` | `getNumFolds()`             Gets the number of folds for cross-validation. |
| `java.lang.String[]` | `getOptions()`             Gets the current settings of the Classifier. |
| `java.lang.String` | `getRevision()`             Returns the revision string. |
| `int` | `getSeed()`             Gets the random number seed. |
| `java.lang.String` | `globalInfo()`             Returns a string describing classifier |
| `java.util.Enumeration` | `listOptions()`             Returns an enumeration describing the available options. |
| `static void` | `main(java.lang.String[] argv)`             Main method for testing this class. |
| `java.lang.String` | `numFoldsTipText()`             Returns the tip text for this property |
| `java.lang.String` | `seedTipText()`             Returns the tip text for this property |
| `void` | `setClassifiers(Classifier[] classifiers)`             Sets the list of possible classifers to choose from. |
| `void` | `setDebug(boolean debug)`             Set debugging mode |
| `void` | `setNumFolds(int numFolds)`             Sets the number of folds for cross-validation. |
| `void` | `setOptions(java.lang.String[] options)`             Parses a given list of options. |
| `void` | `setSeed(int seed)`             Sets the seed for random number generation. |
| `java.lang.String` | `toString()`             Output a representation of this classifier |

**Methods inherited from class weka.classifiers.MultipleClassifiersCombiner**

`getCapabilities`

**Methods inherited from class weka.classifiers.Classifier**

`classifyInstance, forName, makeCopies, makeCopy`

**Methods inherited from class java.lang.Object**

`equals, getClass, hashCode, notify, notifyAll, wait, wait, wait`

**Constructor Detail**

### MultiScheme

```
public MultiScheme()
```


**Method Detail**

### globalInfo

```
public java.lang.String globalInfo()
```

:   Returns a string describing classifier

    :   **Returns:**: a description suitable for displaying in the explorer/experimenter gui

---


### listOptions

```
public java.util.Enumeration listOptions()
```

:   Returns an enumeration describing the available options.

    :   **Specified by:**: `listOptions` in interface `OptionHandler` **Overrides:**: `listOptions` in class `RandomizableMultipleClassifiersCombiner`
    :   **Returns:**: an enumeration of all the available options.

---


### setOptions

```
public void setOptions(java.lang.String[] options)
                throws java.lang.Exception
```

:   Parses a given list of options.


    Valid options are:

    ```
     -X <number of folds>
      Use cross validation for model selection using the
      given number of folds. (default 0, is to
      use training error)
    ```

    ```
     -S <num>
      Random number seed.
      (default 1)
    ```

    ```
     -B <classifier specification>
      Full class name of classifier to include, followed
      by scheme options. May be specified multiple times.
      (default: "weka.classifiers.rules.ZeroR")
    ```

    ```
     -D
      If set, classifier is run in debug mode and
      may output additional info to the console
    ```

    :   **Specified by:**: `setOptions` in interface `OptionHandler` **Overrides:**: `setOptions` in class `RandomizableMultipleClassifiersCombiner`
    :   **Parameters:**: `options` - the list of options as an array of strings **Throws:**: `java.lang.Exception` - if an option is not supported

---


### getOptions

```
public java.lang.String[] getOptions()
```

:   Gets the current settings of the Classifier.

    :   **Specified by:**: `getOptions` in interface `OptionHandler` **Overrides:**: `getOptions` in class `RandomizableMultipleClassifiersCombiner`
    :   **Returns:**: an array of strings suitable for passing to setOptions

---


### classifiersTipText

```
public java.lang.String classifiersTipText()
```

:   Returns the tip text for this property

    :   **Overrides:**: `classifiersTipText` in class `MultipleClassifiersCombiner`
    :   **Returns:**: tip text for this property suitable for displaying in the explorer/experimenter gui

---


### setClassifiers

```
public void setClassifiers(Classifier[] classifiers)
```

:   Sets the list of possible classifers to choose from.

    :   **Overrides:**: `setClassifiers` in class `MultipleClassifiersCombiner`
    :   **Parameters:**: `classifiers` - an array of classifiers with all options set.

---


### getClassifiers

```
public Classifier[] getClassifiers()
```

:   Gets the list of possible classifers to choose from.

    :   **Overrides:**: `getClassifiers` in class `MultipleClassifiersCombiner`
    :   **Returns:**: the array of Classifiers

---


### getClassifier

```
public Classifier getClassifier(int index)
```

:   Gets a single classifier from the set of available classifiers.

    :   **Overrides:**: `getClassifier` in class `MultipleClassifiersCombiner`
    :   **Parameters:**: `index` - the index of the classifier wanted **Returns:**: the Classifier

---


### seedTipText

```
public java.lang.String seedTipText()
```

:   Returns the tip text for this property

    :   **Overrides:**: `seedTipText` in class `RandomizableMultipleClassifiersCombiner`
    :   **Returns:**: tip text for this property suitable for displaying in the explorer/experimenter gui

---


### setSeed

```
public void setSeed(int seed)
```

:   Sets the seed for random number generation.

    :   **Specified by:**: `setSeed` in interface `Randomizable` **Overrides:**: `setSeed` in class `RandomizableMultipleClassifiersCombiner`
    :   **Parameters:**: `seed` - the random number seed

---


### getSeed

```
public int getSeed()
```

:   Gets the random number seed.

    :   **Specified by:**: `getSeed` in interface `Randomizable` **Overrides:**: `getSeed` in class `RandomizableMultipleClassifiersCombiner`
    :   **Returns:**: the random number seed

---


### numFoldsTipText

```
public java.lang.String numFoldsTipText()
```

:   Returns the tip text for this property

    :   **Returns:**: tip text for this property suitable for displaying in the explorer/experimenter gui

---


### getNumFolds

```
public int getNumFolds()
```

:   Gets the number of folds for cross-validation. A number less
    than 2 specifies using training error rather than cross-validation.

    :   **Returns:**: the number of folds for cross-validation

---


### setNumFolds

```
public void setNumFolds(int numFolds)
```

:   Sets the number of folds for cross-validation. A number less
    than 2 specifies using training error rather than cross-validation.

    :   **Parameters:**: `numFolds` - the number of folds for cross-validation

---


### debugTipText

```
public java.lang.String debugTipText()
```

:   Returns the tip text for this property

    :   **Overrides:**: `debugTipText` in class `Classifier`
    :   **Returns:**: tip text for this property suitable for displaying in the explorer/experimenter gui

---


### setDebug

```
public void setDebug(boolean debug)
```

:   Set debugging mode

    :   **Overrides:**: `setDebug` in class `Classifier`
    :   **Parameters:**: `debug` - true if debug output should be printed

---


### getDebug

```
public boolean getDebug()
```

:   Get whether debugging is turned on

    :   **Overrides:**: `getDebug` in class `Classifier`
    :   **Returns:**: true if debugging output is on

---


### getBestClassifierIndex

```
public int getBestClassifierIndex()
```

:   Get the index of the classifier that was determined as best during
    cross-validation.

    :   **Returns:**: the index in the classifier array

---


### buildClassifier

```
public void buildClassifier(Instances data)
                     throws java.lang.Exception
```

:   Buildclassifier selects a classifier from the set of classifiers
    by minimising error on the training data.

    :   **Specified by:**: `buildClassifier` in class `Classifier`
    :   **Parameters:**: `data` - the training data to be used for generating the boosted classifier. **Throws:**: `java.lang.Exception` - if the classifier could not be built successfully

---


### distributionForInstance

```
public double[] distributionForInstance(Instance instance)
                                 throws java.lang.Exception
```

:   Returns class probabilities.

    :   **Overrides:**: `distributionForInstance` in class `Classifier`
    :   **Parameters:**: `instance` - the instance to be classified **Returns:**: the distribution for the instance **Throws:**: `java.lang.Exception` - if instance could not be classified successfully

---


### toString

```
public java.lang.String toString()
```

:   Output a representation of this classifier

    :   **Overrides:**: `toString` in class `java.lang.Object`
    :   **Returns:**: a string representation of the classifier

---


### getRevision

```
public java.lang.String getRevision()
```

:   Returns the revision string.

    :   **Specified by:**: `getRevision` in interface `RevisionHandler` **Overrides:**: `getRevision` in class `Classifier`
    :   **Returns:**: the revision

---


### main

```
public static void main(java.lang.String[] argv)
```

:   Main method for testing this class.

    :   **Parameters:**: `argv` - should contain the following arguments: -t training file [-T test file] [-c class index]


---


|  |  |  |
| --- | --- | --- |
| **Overview** \| **Package** \| **Class** \| **Tree** \| **Deprecated** \| **Index** \| **Help** \| **Weka's home** | |  |
| **PREV CLASS**   **NEXT CLASS** | **FRAMES**    **NO FRAMES**     **All Classes** |  |
| SUMMARY: NESTED \| FIELD \| CONSTR \| METHOD | DETAIL: FIELD \| CONSTR \| METHOD |  |


---
